# Two new species of *Euptychia* Hübner, 1818 from the upper Amazon basin (Lepidoptera, Nymphalidae, Satyrinae)

**DOI:** 10.3897/zookeys.541.6297

**Published:** 2015-12-01

**Authors:** Andrew F. E. Neild, Shinichi Nakahara, Thamara Zacca, Steven Fratello, Gerardo Lamas, Jean-François Le Crom, Diego R. Dolibaina, Fernando M. S. Dias, Mirna M. Casagrande, Olaf H. H. Mielke, Marianne Espeland

**Affiliations:** 1 Scientific Associate, The Natural History Museum, Life Sciences Dept., Cromwell Rd., London SW7 5BD, UK; 2 McGuire Center for Lepidoptera and Biodiversity, Florida Museum of Natural History, University of Florida, Gainesville, FL 32611, USA; 3 Departamento de Zoologia, Laboratório de Estudos de Lepidoptera Neotropical, Universidade Federal do Paraná – UFPR, Caixa Postal 19020, 81531-980, Curitiba, Paraná, Brasil; 4 11 First St., W. Islip, NY 11795, USA; 5 Museo de Historia Natural, Universidad Nacional Mayor de San Marcos, Apartado 14-0434, Lima-14, Peru; 6 Calle 61, N° 37-31, Bogotá, Colombia

**Keywords:** Euptychiina, Neotropics, South America, Brazil, Colombia, Peru, Venezuela, mitochondrial DNA COI barcode

## Abstract

Two new species of *Euptychia* Hübner, 1818 are described from the upper Amazon basin: *Euptychia attenboroughi* Neild, Nakahara, Fratello & Le Crom, **sp. n.** (type locality: Amazonas, Venezuela), and *Euptychia sophiae* Zacca, Nakahara, Dolibaina & Dias, **sp. n.** (type locality: Acre, Brazil). Their unusual facies prompted molecular and phylogenetic analyses of one of the species resulting in support for their classification in monophyletic *Euptychia*. Diagnostic characters for the two species are presented based on wing morphology, wing pattern, presence of androconial patches on the hindwing, and genitalia. Our results indicate that the projection of the tegumen above the uncus, previously considered a synapomorphy for *Euptychia*, is not shared by all species in the genus. The adults and their genitalia are documented, and distribution data and a map are provided.

## Abstract (pt)

Duas novas espécies de *Euptychia* Hübner, 1818 são descritas da bacia do alto Rio Amazonas: *Euptychia attenboroughi* Neild, Nakahara, Fratello & Le Crom, **sp. n.** (localidade tipo: Amazonas, Venezuela), *Euptychia sophiae* Zacca, Nakahara, Dolibaina & Dias, **sp. n.** (localidade tipo: Acre, Brasil). Suas aparências não usuais levaram a análises molecular e filogenética de uma das espécies que sustentam sua classificação como *Euptychia*. São apresentados caracteres diagnósticos para as duas espécies baseados na morfologia e no padrão de coloração de suas asas, presença de manchas androconiais nas asas posteriores e suas genitálias. Os resultados indicam que a projeção do tegume sobre o unco, previamente considerada uma sinapomorfia para *Euptychia*, não é compartilhada por todas as espécies do gênero. São ilustrados os adultos e suas genitálias, bem como dados e mapa de distribuição.

## Abstract (es)

Se describen dos nuevas especies de *Euptychia* Hübner, 1818 para la cuenca alta del Río Amazonas: *Euptychia attenboroughi* Neild, Nakahara, Fratello & Le Crom, **sp. n.** (localidad típica: Amazonas, Venezuela) y *Euptychia sophiae* Zacca, Nakahara, Dolibaina & Dias, **sp. n.** (localidad típica: Acre, Brasil). Sus apariencias inusuales motivaron el análisis molecular y filogenético de una de las especies, lo cual sustenta su clasificación como *Euptychia*. Se presentan caracteres diagnósticos para las dos especies basados en la morfología y patrón de coloración alar, presencia de parches androconiales en las alas posteriores y sus genitales. Los resultados indican que la proyección del tegumen sobre el uncus, previamente considerada una sinapomorfía para *Euptychia*, no es compartida por todas las especies del género. Se ilustran los adultos y sus genitales, y se proporcionan datos y mapa de distribución.

## Introduction

The nymphalid subtribe Euptychiina (Satyrinae: Satyrini) is one of the most poorly known butterfly groups. More than 400 predominantly Neotropical species in some 50 genera are recognised within the subtribe and many taxa remain undescribed (Lamas 2004; pers. obs.). However, generic classification of species within the subtribe is confused mainly because of a lack of clear morphological characters and morphological homogeneity (Peña and Lamas 2005). In addition, their drab coloration has probably contributed to this group being ignored by many lepidopterists in the field. Forster (1964) described 33 euptychiine genera which are mostly accepted today, but provided few reliable diagnostic characters for these taxa. As a result, placement of species in these genera is often tentative: by way of example, genera such as *Magneuptychia* Forster, 1964, *Cissia* Doubleday, 1848, and *Splendeuptychia* Forster, 1964 are recovered as polyphyletic or paraphyletic in recent phylogenetic analyses (e.g. Peña et al. 2010), indicating the confused generic-level taxonomy. On the other hand, the genus *Euptychia* Hübner, 1818 is relatively well known compared to other euptychiine genera, and is morphologically defined by the posterior projection of the tegumen above the uncus in the male genitalia. This character is considered to be a good synapomorphy (Freitas et al. 2012) and has been used to classify recently described *Euptychia* species (Neild et al. 2014, Nakahara et al. 2014). Many other described and undescribed *Euptychia* species also possess this character (Nakahara, unpub.), and its presence even contributed to the inclusion of *Caenoptychia boulleti* Le Cerf, 1919 in *Euptychia* (Freitas et al. 2012). However, we here describe two new species of *Euptychia* which lack this posterior projection of the tegumen. The placement of these two species in *Euptychia* is supported by molecular data, which are presented in this paper, and by many alternative possible diagnostic characters for the genus, which are discussed.

## Methods

### Morphology

Comparison of the morphology of the two new species was made with other *Euptychia* specimens in the collections listed below (museum acronyms are from Heppner and Lamas 1982):

AMNH American Museum of Natural History, New York, USA

AN Andrew Neild collection, London, UK

BMNH The Natural History Museum, London, UK

DZUP Coleção Entomológica Padre Jesus de Santiago Moure, Universidade Federal do Paraná, Curitiba, Brazil

ICN-MHN-L Instituto de Ciencias Naturales, Colección de Lepidoptera, Universidad Nacional de Colombia, Bogotá, Colombia

MGCL McGuire Center for Lepidoptera and Biodiversity, Florida Museum of Natural History, Gainesville, USA

MUSM Museo de Historia Natural, Universidad Nacional Mayor de San Marcos, Lima, Peru

Type specimens of *Euptychia* species in the BMNH were also checked, as well as photographs of additional taxa on the website “Butterflies of America” (available online in Warren et al. 2015). In addition, recent works on French Guianan *Euptychia* by Brévignon (2005, 2008) and Brévignon and Benmesbah (2012) were consulted, and our taxa were discussed with the authors of those papers. Wings were diaphanised using standard techniques for Lepidoptera. The abdomens of the Venezuelan pair of *Euptychia attenboroughi* sp. n. and one of the Brazilian males of *Euptychia sophiae* sp. n. were dissected to observe genital structures. Abdomens were dissected using standard techniques, with adult abdomens being soaked in hot 10% KOH solution for 3–10 minutes, dissected and subsequently stored in microvials in glycerine. External morphology and dissections were studied using stereomicroscopes and photographed using digital cameras. The terminology for genital and abdominal structures follows Scoble (1992), with additional detail derived from Klots (1956). Our use of the term “vinculum” agrees with Austin and Mielke (2008). Nomenclature for wing venation corresponds to that of Miller (1970: 46), and wing areas to Neild (2008: fig. 1.2).

The following abbreviations are used:

FW forewing

HW hindwing

D dorsal

V ventral

### Molecular and phylogenetic analysis

A leg from the Brazilian male paratype (DZ 29.578) of *Euptychia sophiae* sp. n. was used to obtain a DNA sequence for cytochrome c oxidase subunit I (COI) and elongation factor-1 alpha (ef1a). We extracted DNA using Qiagen’s DNeasy Blood & Tissue Kit, following the protocol and using a final elution volume of 50 µl. Primers LCO_nym (forward, 5'TTTCTACAAATCATAAAGATATTGG 3’) and HCO_nym (reverse, 5'TAAACTTCAGGGGTGACCAAAAAATCA 3’) were used to amplify COI. Elongation factor-1 alpha was amplified by using primer pairs Ef44 (forward, 5'GCYGARCGYGARCGTGGTATYAC 3’), and EfrcM4 (reverse, 5'ACAGCVACKGTYTGYCTCATRTC 3’); however, since this primer pair failed to amplify ef1a, the following primer pair was used to amplify short fragments of ef1a: Ef44 (forward, 5'GCYGARCGYGARCGTGGTATYAC 3’) and Monica (reverse, 5'CATRTTGTCKCCGTGCCARCC 3’) (Monteiro and Pierce 2001). All PCR reactions were conducted in a 25 µl volume comprising 1 µl of template DNA, 9.5 µl ddH2O, 1 µl of each primer (10 µM), and 12.5 µl Omega 2x Taq Mastermix (Omega Bio-tek, Norcross, GA, USA) (5 U/µl). Reaction conditions were as follows: for COI, 1 min at 94 °C, followed by 5 cycles of 30 s at 94 °C, 40 s at 45 °C, 1 min at 72 °C, followed by 35 cycles of 30 s at 94 °C, 40 s at 51 °C, 1 min at 72 °C, followed by final elongation for 5 min at 72 °C; for Ef1a, 35 cycles of 1 min at 95 °C, 1 min at 58 °C, and 1 min at 72 °C, followed by final extension for 5 min at 72 °C; for short fragment of Ef1a, 1 min at 95 °C, 40 cycles of 30 s at 94 °C, 40 s at 58 °C, and 1 min at 72 °C, followed by final extension for 5 min at 72 °C. PCR products were checked on 1.2% agarose gels stained with ethidium bromide. Purification and sequencing were completed at the Interdisciplinary Center for Biotechnology Research (ICBR) at the University of Florida. Two sequences were uploaded to GenBank (KR818703, KR818706) and were analysed with 8 other *Euptychia* species and 2 outgroup taxa from GenBank (see Table 1).

**Table 1.** GenBank accession numbers for sequences used in this study.

Genus	Species	Voucher code	COI	EF1a
*Euptychia*	*enyo*	CP06-73	GQ357205	GQ357275
*Euptychia*	sp. 2	CP01-33	DQ338794	DQ338937
*Euptychia*	*westwoodi*	DNA96-005	AY508543	AY509069
*Euptychia*	*boulleti*	PM17-01	JQ639284	JQ639285
*Euptychia*	sp. 6	CP04-55	DQ338796	DQ338939
*Euptychia*	*sophiae*	DZ 29.578	KR818703	KR818706
*Euptychia*	*picea*	DNA99-036	AY508542	AY509068
*Euptychia*	sp. 5	CP01-53	DQ338795	DQ338938
*Euptychia*	sp. 7	CP02-58	GQ357206	DQ338940
*Magneuptychia*	*fugitiva*	CP01-18 / DNA99-008	GU205845	AY509078
*Papilio*	*machaon*	BC ZSMLep 27060 / NA	GU707119	EF485106

Sequences were aligned using MAFFT v. 7.107 (Katoh et al. 2002). Best-fitting models and partitioning schemes were jointly selected using PartitionFinder v. 1.1.1. (Lanfear et al. 2012). The program was run twice, once with the models available in MrBayes 3.2.3 (Ronquist et al. 2012) and once with those in RAxML v. 8.1.11 (Stamatakis 2006). The selected partitioning schemes and models can be found in Table 2.

**Table 2.** Partitioning schemes and substitution models determined by Partitionfinder.

Partition	Best model MrBayes	Subset partitions BEAST	Best model RAxML	Subset partitions RAxML
1	GTR+I	COI position 1	GTR	COI position 1
2	HKY+I	COI position 2	GTR	COI position 2
3	HKY+G	COI position 3	GTR	COI position 3
4	GTR	EF1a position 1	GTR	EF1a position 1
5	HKY	EF1a position 2	GTR	EF1a position 2
6	GTR+G	EF1a position 3	GTR	EF1a position 3

Phylogenies were inferred using maximum likelihood (ML) and Bayesian inference on the concatenated data set of 1041bp. ML analyses were run using RAxML v 8.1.11 (Stamatakis 2006, Stamatakis et al. 2008) with the GTR model for all partitions (as selected by PartitionFinder) and 1000 rapid bootstrap replicates followed by 200 thorough maximum likelihood searches with joint branch length optimization. Bayesian analyses were run in MrBayes 3.2.3. Analyses were run twice for 10 million generations with trees sampled every 1000 generations with the temperature parameter set to 0.15. Model parameters were unlinked across data partitions, and the prior for rate variation among partitions was set to variable. The first 25% of the samples were discarded as burn-in. Convergence was checked in Tracer 1.6 (Rambaut and Drummond 2007). RAxML and MrBayes were run on the CIPRES cluster (Miller et al. 2010).

## Results

**Figure 1.**
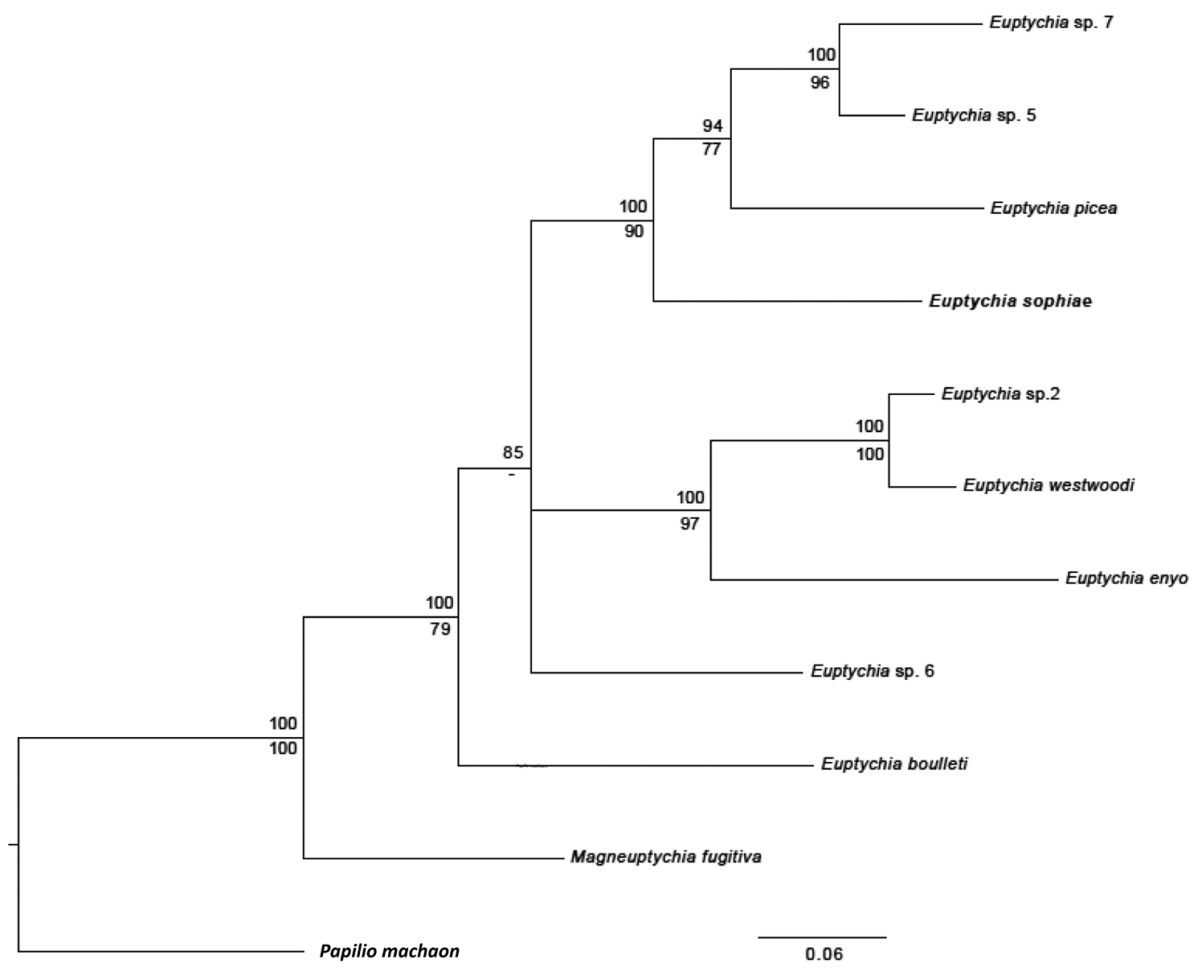
Bayesian phylogeny of *Euptychia* based on one mitochondrial (COI) and one nuclear (EF1-a) gene. Posterior probabilities are listed above and bootstrap values below branches. A dash denotes bootstrap support lower than 50%. (*Euptychia attenboroughi* is not included in the analysis – see text for details.)

According to ML and Bayesian inference based on barcoding region and EF1a, *Euptychia sophiae* sp. n. is sister to the clade consisting of *Euptychia picea* Butler, 1867 and two undescribed species. Unlike Freitas et al. (2012), *Euptychia boulleti* (Le Cerf, 1919) is here placed as sister to the clade with *Euptychia enyo* Butler, 1867, *Euptychia westwoodi* Butler, 1867, and an undescribed species in the ML tree, although with relatively low support. However, in the Bayesian tree (Fig. 1), this species appears as sister to the rest of *Euptychia*. Thus in this tree the ancestor of *Euptychia* (minus *Euptychia boulleti*) has no bootstrap support since this node does not exist in the ML tree. Placement of *Euptychia sophiae* sp. n. in monophyletic *Euptychia* is supported by both analyses. The results of this molecular analysis are supported by the ML analysis in Nakahara et al. (2015), which included over 150 euptychiine and other satyrine taxa and recovered the genus *Euptychia* as monophyletic and including *Euptychia sophiae* (as *Euptychia*_n_sp).

## Taxonomy

Diagnostic morphological characters seen in these two new species which appear to be unique among the genus *Euptychia* include: (i) presence of a single signum in female *Euptychia attenboroughi* sp. n. (no female is known for *Euptychia sophiae* sp. n.); (ii) lamella antevaginalis unsclerotised around the ostium bursae of female *Euptychia attenboroughi* sp. n. (sclerotised in all other *Euptychia* examined), except on the ventral surface around the neck of the ostium bursae; (iii) reduced third segment of labial palpus of female *Euptychia attenboroughi* sp. n. (Fig. 5); (iv) presence of a prominent ventral hindwing androconial patch in male *Euptychia sophiae* sp. n. None of these characters are known in any other species of *Euptychia* we have examined and are considered to be diagnostic for *Euptychia attenboroughi* sp. n. (states i, ii and iii) and *Euptychia sophiae* sp. n. (state iv). For diagnostic characters for *Euptychia attenboroughi* sp. n. and *Euptychia sophiae* sp. n., consult the diagnosis for each species.

### Euptychia attenboroughi

Taxon classificationAnimaliaLepidopteraNymphalidae

Neild, Nakahara, Fratello & Le Crom sp. n.

http://zoobank.org/FA31E137-39EC-4B26-B9E8-48E7F4032773

**Figure 2.**
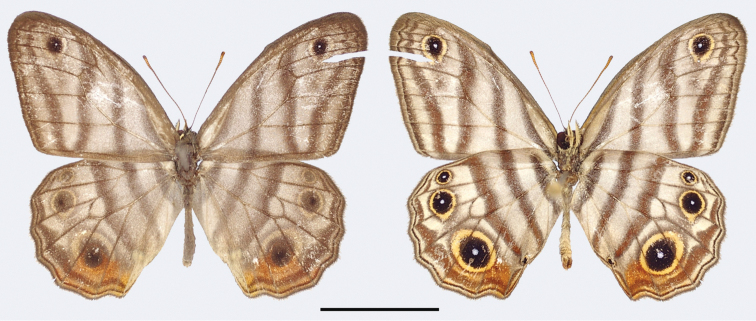
*Euptychia attenboroughi* sp. n. holotype male, dorsal (left) and ventral (right). FW length: 18.0 mm. BMNH collection. Photos by Andrew Neild, Trustees of the Natural History Museum, London. Scale bar: 10 mm.

**Figure 3.**
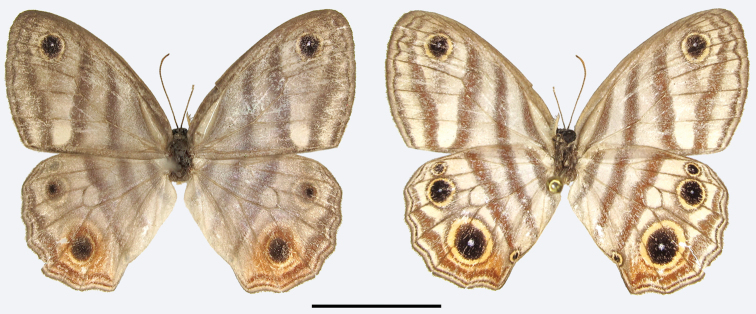
*Euptychia attenboroughi* sp. n. paratype female, dorsal (left) and ventral (right). FW length: 16.0 mm. AN collection. Photos by Andrew Neild. Scale bar: 10 mm.

**Figure 4.**
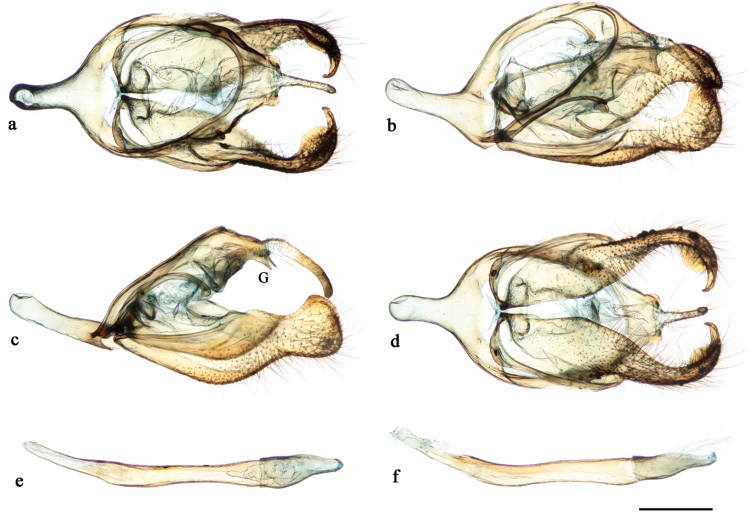
*Euptychia attenboroughi* sp. n. Genitalia of holotype male: **a** dorsal view **b** dorso-lateral view **c** lateral view (“G” indicates fused gnathos) **d** ventral view **e** aedeagus, dorsal view, and **f** lateral view. BMNH collection. Photos by Andrew Neild, Trustees of the Natural History Museum, London. Scale bar: 0.5 mm.

**Figure 5.**
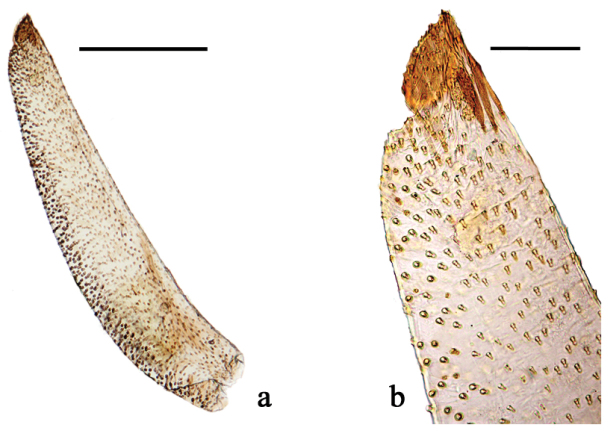
*Euptychia attenboroughi* sp. n. Left labial palpus of paratype female: **a** lateral view of second and third segment **b** detail of second and third segment. AN collection. Photos by Andrew Neild. Scale bar: 0.5 mm (**a**), 0.1 mm (**b**)

**Figure 6.**
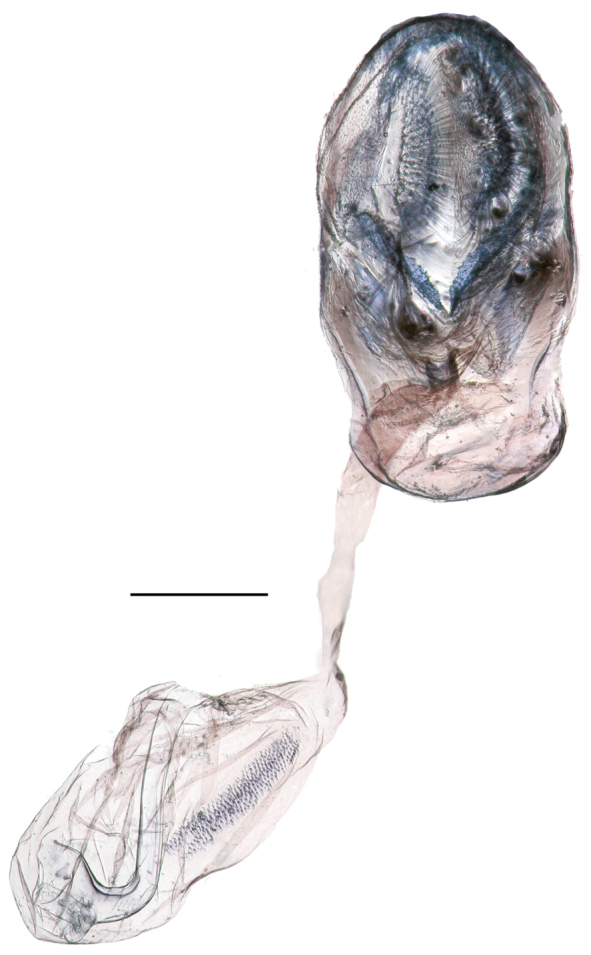
*Euptychia attenboroughi* sp. n. Genitalia of paratype female: postero-ventral view. AN collection. Photos by Andrew Neild. Scale bar: 0.5 mm.

**Map 1.**
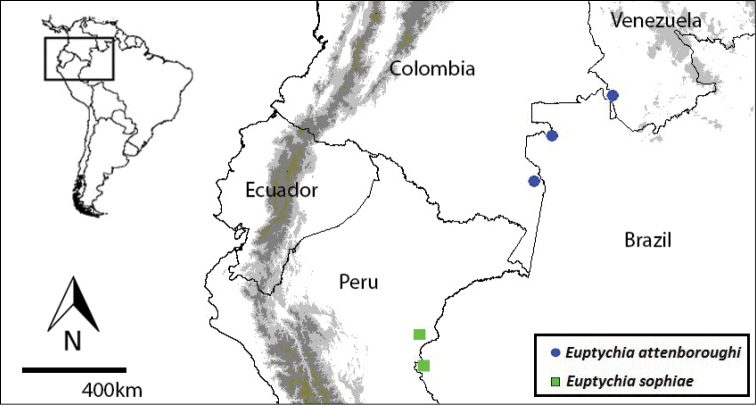
Distribution of *Euptychia attenboroughi* sp. n. (blue circles) and *Euptychia sophiae* sp. n. (green squares).

Figs 2 , 3 , 4 , 5 , 6 ; Map 1

#### Type material.

Holotype male with the following labels (separated by transverse bars): HOLOTYPE / VENEZUELA – *Amazonas*: San Carlos de Rio Negro to Solano track, km. 3 [approximately 1°55'N 67°1'W], 5-17 III 94, 100m elev., Andrew Neild Collection / Brit. Mus. 1994-298 / BMNH(E) #1054424 (BMNH).

#### Paratypes.

VENEZUELA – *Amazonas*: 1 female, same locality data as holotype / (Neild Prep. Genital Vial No. 274) (AN); BRAZIL – *Amazonas*: 1 female, Janarete [= Jauaretê] (approximately 0°36'N 69°11'W), IX, 1943 / W. Praetorius Coll. Donor Frank Johnson (AMNH); COLOMBIA – *Vaupés*: 1 male, Camino a mina “La Libertad”, camino a caño grande “Pescadero”, en bosque amazónico [approximately 1°01'N 69°45'W, north of Chorro La Libertad, *fide* Jaime Pinzón], 10:30 am, 290 m, 25-Agosto-1993, Col: G. Fagua (ICN-MHN-L); 1 female, Serranía de Taraira; camino a mina “La Libertad”, camino a mina “Marulanda”; en rastrojo [approximately 1°01'N 69°45'W, north of Chorro La Libertad, *fide* Jaime Pinzón], 2:45pm, 290 m, 8 agosto 1993, G. Fagua (ICN-MHN-L); *No data*: 1 male, (genital vial # SN-15-59) (MGCL).

#### Diagnosis.

Differs from males of its congener *Euptychia sophiae* sp. n. as follows (no females of *Euptychia sophiae* sp. n. are known, but we expect characters indicated with an asterisk (*) will serve to differentiate this sex): (1) FW more produced apically, with outer margin straighter or more concave, and outer margin at an angle away from the central line of the body (nearly parallel in *Euptychia sophiae* sp. n.); (2) dorsally with prominent orange scaling on the posterior DHW on and near the tornus* (*Euptychia sophiae* sp. n. lacks this orange scaling); (3) VHW submarginal band does not increase in width at the tornus (increases substantially for *Euptychia sophiae* sp. n.); (4) small ocellus at the anal margin of the VHW median band larger*; (5) androconial patch on VHW pale and barely visible, whereas prominent on *Euptychia sophiae* sp. n.; (6) gnathos projecting nearly parallel to the uncus (projecting vertically in *Euptychia sophiae* sp. n.); (7) distal one-fourth of the valva broad in lateral view (narrow in *Euptychia sophiae* sp. n.); (8) aedeagus straighter, less curved.

The female resembles several species in the genus *Euptychia*, especially *Euptychia marceli* Brévignon, 2005, but is distinguishable on the VHW through the unique combination of a very large and ovoid (not round) ocellus in Cu_1_-Cu_2_ bordered on its tornal side by orange scaling, and by the presence of a tiny ocellus on the anal margin at the posterior end of the median brown band (this last found rarely on some specimens of *Euptychia marceli* Brévignon, 2005). It can also be differentiated from all other known *Euptychia* females by the three characters (i, ii, and iii) elucidated at the beginning of the Taxonomy section. The male somewhat resembles a few taxa currently in *Chloreuptychia* Forster, 1964 (see Warren et al. 2015), with its wing shape and greyish translucence, but differs by the absence of a bluish sheen on either wing surface, presence of orange HW tornal scaling on both wing surfaces, presence of a small VHW ocellus at the anal margin, absence of orange- and silver-lined ovoid VHW markings in M_2_-M_3_ and M_3_-Cu_1_, and by the single silvery-white pupils in the VHW ocelli (double in most *Chloreuptychia* species).

#### Description.

MALE (Fig. 2):

Forewing length. 17.0–18.0 mm (n = 3) (holotype = 18 mm).

Head. Frons brownish; postgenal area light brown.

Antennae. Naked, orange-brown, darker dorsally, clubs browner with orange tip, 7–8 mm long.

Eyes. Dark brown, sparsely hairy; creamy-grey scales dorsally and laterally along posterior edge of eyes.

Palpi. Covered by long creamy-grey hair-like modified scales dorso-laterally, ventrally with long fine hair-like modified scales projecting like a Mohican, mostly black along outer margin, but interior wall of modified scales creamy-grey. Mohican highest in centre, gradually reducing anteriorly and posteriorly, and anteriorly reduced to a pointed tuft. First segment covered with black scales dorsally, black and white hair-like modified scales ventrally, second segment covered with short white hair-like modified scales and white scales laterally, black scales distal one-third of dorsal surface, ventrally adorned with long black and white hair-like modified scales 3–4 times as long as segment width, second segment slightly longer than eye diameter, third segment covered with black scales dorsally and ventrally, creamy-white hair-like scales laterally, about one-seventh of second segment in length.

Thorax. Covered in long light grey hair-like modified scales.

Legs. Greyish. Foreleg tarsus about 2/5 of tibia in length, femur about 2/3 of tibia in length; tibial spurs absent on midleg and hindleg.

Abdomen. Eighth tergite and sternite well developed, apparently as equally sclerotized as other tergites and sternites, but weakly sclerotized towards posterior end.

Androconia patches. Modified wing scales, presumed to be androconia, present on either side of vein 2A on the HW at the base of the dark median band on the dorsal and ventral surface; visible on the dorsal surface as two tiny ovoid pale greyish-brown patches approximately 0.5 mm long, and on the ventral surface as a strip approximately 2.0 mm long in space 2A–3A; these patches, best viewed using backlighting, are homologous with the dorsal and ventral androconia patches of *Euptychia sophiae* sp. n.

Wing venation. FW recurrent vein present, approximately 1.75 mm long; FW vein Cu not swollen at base; HW with humeral vein barely visible, very short (approximately 0.6 mm), curved anteriorly towards the costal lobe.

Wing shape. FW costa gently convex to apex, apex relatively pointed, outer margin straight, or slightly concave between M_3_ and Cu_2_, and angling about 20 degrees away from the central line of the body, inner margin almost straight; HW costa lobed in basal area then gently concave to apex at Sc+R_1_, apex rounded, outer margin scalloped, anal margin concave near tornus, basally convex.

DFW. Both wings slightly and variably translucent with greyish-brown to chestnut brown ground colour; fringes greyish-brown to brown; four diffuse dark brown to chestnut bands crossing from the costal to the anal margin, the first basal and barely visible, mostly ghosting through the slightly translucent wing from the ventral surface, centrally wide but tapering to a point anteriorly and posteriorly, the second submedian, wider, nearly straight and better defined, crosses the mid discal cell from mid costa to four tenths along the inner margin, the third median slightly wavy strongly defined and the widest, crosses from the costa near the discocellular veins, which it traverses, reaching to seven tenths distance along the inner margin, the fourth begins near the apex where it is narrow and very sinuous, runs parallel to the outer margin and curved in each interspace down to M_3_ and then angles without curves in towards the body and widens reaching the submarginal area of Cu_1_, then again running parallel to the outer margin down to the inner margin near the tornus; the margin with a very fine dark brown line running parallel to the outer margin, beginning at the apex, incurved in each interspace to M_3_, then straight to the tornus; a white-pupilled black subapical ocellus in the centre of M_1_-M_2_, touching M_2_ but not quite reaching M_1_.

DHW. Four diffuse dark brown to chestnut bands crossing from the costal to the anal margin, the first basal, mostly ghosting through the slightly translucent wing from the ventral surface, the second submedian also ghosting through, nearly straight, crosses the mid discal cell from mid costa to half distance along the anal margin, the third median and slightly wavy, in some specimens (two of the three males) curved inwards in the distal discal cell, better defined with dorsal scaling (less ghosting) and the widest, crosses from the costa two-thirds towards the apex, almost reaching 2A two-thirds along its length, and not passing to the anal margin, the fourth submarginal begins near the apex where it is narrow, runs parallel to the outer margin (curved in each interspace), gradually thickening, widest in M_2_-M_3_ (where the basal edge points inward) and M_3_-Cu_1_, and then thinning gradually to just reach 2A near the tornus; the margin with a very fine clearly defined dark brown line running parallel to the outer margin, beginning at Sc+R_1_, incurved in each interspace to the tornus and entering the anal lobe to 3A; three ventral ocelli visible through the slightly translucent wings, the dark circular areas showing through from the ventral surface in cells Cu_1_-Cu_2_ and M_1_-M_2_ covered with small spheroid areas of diffuse very dark brown dorsal scaling, the latter very small, the former roughly half the diameter of the black ventral “iris”; orange scaling on the distal side of the large ocellus in space Cu_1_-Cu_2_ continues to the outer margin and in the same areas of Cu_2_-2A.

VFW. Both wings slightly translucent with pale greyish-brown ground colour; fringes greyish-brown; one very thin well-defined submarginal dark brown to chestnut band and four more diffuse dark brown to chestnut basal bands, submedian, median and postmedian crossing from the costal to the anal margin, as described for the dorsal surface; a silvery-white-pupilled black subapical ocellus in the centre of space M_1_-M_2_, touching M_2_ but not quite reaching M_1_, circled by a gold ring with a thin grey-brown outer edge which enters R_5_-M_1_; in M_2_-M_3_ the grey-brown edge of the ocellar ring breaks open and the yellow area spills posteriorly into the centre of the interspace; in one specimen (MGCL) there is an additional tiny ocellus in Cu_1_-Cu_2_ with a reddish-brown “iris” (of the same colour as the transverse bands), a tiny pupil (or merely missing scales?), and an outer ring of the same colour as the background pale-grey-brown, surrounded by an indistinct scattering of brownish scales that define the edge of the outer pale ring.

VHW. One very thin well-defined marginal dark brown band and four more diffuse dark brown to chestnut basal bands, submedian, median, and marginal crossing from the costal to the anal margin, as described for the dorsal surface, but the submarginal and marginal bands continue from the tornus along the anal margin to the base of the chestnut median band, while the marginal band (only) continues to the base of the submedian band; a very small ocellus on the anal margin at the base of the median brown band composed of a large black subovoid “iris”, a narrow golden outer ring, and a narrow dark brown border; three postmedian ocelli, composed of a single small silvery-white pupil, a large black spheroid or ovoid “iris”, and a narrow golden outer ring enclosed in a narrow grey-brown border; the smallest of these ocelli is spheroid, entirely within Rs-M_1_, not touching either vein, the second, nearly twice as large, spheroid and occupying the full width of M_1_-M_2_, with the grey-brown outer border just touching M_1_, and the black “iris” just spilling over into M_2_-M_3_, and in the same way as on the VFW, with the outer posterior border broken open in M_2_-M_3_ and the golden outer ring protruding into the centre of the interspace, where a poorly defined wide suffused band of greyish brown scales links this and the third largest ocellus, ovoid, over three times the diameter of the first at its widest point (parallel to the outer margin), with the black “iris” fully occupying Cu_1_-Cu_2_, spilling into M_3_-Cu_1_ but just touching Cu_2_, the golden outer ring double the width of the other two ocelli, reaching the centre of M_3_-Cu_1_, and entering Cu_2_-2A, surrounded by a grey brown ring with indistinct edges; yellow-orange scaling on the distal side of the large ocellus in space Cu_1_-Cu_2_ continues slightly into M_3_-Cu_1_ and fully to the submarginal band of Cu_1_-Cu_2_ with more extensive orange scaling in the distal quarter of Cu_2_-2A just spilling over 2A onto the anal lobe.

Male genitalia (Fig. 4). Tegumen dorsally flattened, ventral edges concave, posterior margin projecting to form a short gnathos fused to the tegumen (approximately one-fifth length of uncus) almost parallel to uncus, but slightly ventrad, somewhat trapezoid in dorsal view; uncus anteriorly hairy, rather narrow and long, posterior tapered and slightly hooked in lateral view, evenly wide in dorsal view; ventral arms of tegumen fused to anterior margin of tegumen; appendices angulares absent; saccus slightly longer than uncus, dorsal arms of saccus combined with ventral arms from tegumen; valva sparsely hairy, basal three-fourths vaguely trapezoidal, distal one-fourth rounded, distal half of valva in dorso-ventral view resembles propodus of a lobster, but without the dactylus; aedeagus tubular, in lateral view rather straight, slightly broadening anterior portion which opens anterodorsally, posterior one third of aedeagus narrow, slightly bent upwards, cornuti absent.

FEMALE (Fig. 3):

Forewing length 16.0–17.0 mm (n = 3). Similar to male except as follows: FW shape not elongate, subtriangular, with distinctly convex outer margin; HW shape similar to male, but slightly less elongate; dorsal surface with all the same dark semi-translucent bands but the basal, submedian, and median bands with more scales present dorsally; the DHW dark circular areas showing through from the ventral surface in cells Cu_1_-Cu_2_ and M_1_-M_2_ almost covered with large spheroid areas of diffuse very dark brown, nearly black, dorsal scaling; the large subtornal ocellus entirely encircled by diffuse orange scaling, and distally by a half-circle of golden scales homologous with the ventral outer ring of this colour; ventrally there are no obvious differences.

Female genitalia (Fig. 6). One Venezuelan female was examined (Neild Prep. Genital Vial No. 274). Lamella antevaginalis sclerotized; area around lamella antevaginalis not sclerotized; very basal side of 8^th^ abdominal segment slightly sclerotized and somewhat ring-like at basal side of 8^th^ abdominal segments; ductus bursae membranous; ductus seminalis located near ostium bursae; corpus bursae oval in dorsal view, with one relatively thick signum.

#### Etymology.

We name this butterfly to honour the great English naturalist, author, and TV presenter, Sir David Attenborough, in gratitude for opening the eyes and hearts of millions to the natural world through his inspiring and edifying work. To prevent any future ambiguity, the name *attenboroughi* is considered to be a Latinised male noun in the genitive case.

#### Distribution, behaviour and habitat.

The six specimens known to date were all collected within 500 kms of each other in the north-west of the upper Amazon basin, representing a very restricted distribution. It is impossible with such a small sample size to draw any concrete conclusions, but we hypothesise that this species is restricted to suitable habitat to the north of the Amazon river, and that its sibling species occurs only to the south, although a limited area of sympatry may exist. One of the senior authors first collected specimens of this new species in 1994 while conducting field work for the *Butterflies of Venezuela* book series (Neild 1996, Neild 2008) in south-western Venezuela. The pair that he collected were settled on low vegetation along a path inside tropical evergreen forest and were netted immediately, before any further observations could be made. One of the Colombian specimens was collected in similar habitat at 10:30 a.m., while the female was captured at 2:45pm outside the forest in scrubby secondary vegetation (*“rastrojo”*). The type series were all found at low elevations from about 100 m to almost 300 m above sea level. The two Venezuelan specimens were collected in the first half of March, during an especially strong dry season which drastically reduced butterfly numbers and species diversity. The Colombian and Brazilian specimens were taken in August and September, months that are also typically among the least wet of the year. The species is evidently very rarely collected, but this may not reflect reality in the field; rather its perceived scarcity may simply be the result of its apparently highly restricted distribution in an area of the Amazon basin that has been, and still is, very little explored.

#### Host plant.

Unknown.

### Euptychia sophiae

Taxon classificationAnimaliaLepidopteraNymphalidae

Zacca, Nakahara, Dolibaina & Dias sp. n.

http://zoobank.org/F81B7816-4C91-4A9B-9105-AD1F3D9F603C

Figs 7 , 8 ; Map 1

#### Type material.

Holotype male with the following labels (separated by transverse bars): /Holotypus/ Brasil, Acre, Mâncio Lima, P[ar]q[ue] Nac[ional] Serra Do Divisor, Porção Norte, 7°26'50"S 73°39'52"W 200-400 m 10-21-IX-2011, D. Dolibaina & D. Moura Leg. / DZ 29.579/ (DZUP).

#### Paratype.

1 male, same data as holotype, except: DZ 29.578 (DZUP); PERU – *Loreto*: 1 male, 45 km. E. de Monte Alegre, Rio Trapiche [rec. Tapiche], 73°47'39.51"S 6°22'58.43"W [*sic* – W and S are transposed], 19–20.ii.2009, 183m, A. Garcia *leg.* (MUSM).

#### Diagnosis.

See relevant section for *Euptychia attenboroughi*.

#### Description.

MALE (Fig. 7):

Forewing length 18–19 mm (n = 3) (holotype = 18 mm).

Head. Brown. Postgenae with creamy-grey scales.

Antennae. Naked, brown, darker dorsally, clubs browner with orange tip.

Eyes. Dark brown, sparsely hairy. Creamy-grey scales dorsally and laterally along posterior edge of eyes.

Palpi. Covered by long creamy-grey hair-like modified scales dorso-laterally, ventrally with long fine hair-like modified scales projecting like a Mohican, mostly black along outer margin, but interior wall of modified scales creamy-grey. Mohican highest in centre, gradually reducing anteriorly and posteriorly, and anteriorly reduced to a pointed tuft. First segment covered with brown scales dorsally, black and creamy hair-like modified scales ventrally, second segment covered with short creamy hair-like modified scales and light brown scales laterally, brown scales distal one-third of dorsal surface, ventrally adorned with long black and creamy hair-like modified scales 3–4 times as long as segment width, second segment slightly longer than eye diameter, third segment covered with brown scales dorsally and black scales ventrally, creamy hair-like scales laterally, about one-seventh of second segment in length.

Thorax. Uniformly covered by dark grey-brown hair-like scales.

Legs. Greyish. Foreleg tarsus more than half-length of tibia, femur about 2/3 of tibia in length; tibial spurs absent on midleg and hindleg.

Abdomen. Eighth tergite and sternite well developed, apparently as equally sclerotized as other tergites and sternites, but weakly sclerotized towards posterior end.

Androconial patches. Two small (< 1.0 mm) pale grey androconial patches on DHW, barely separated by 2A, located at its distal one third; patch in cell 2A–3A prominent; patch in cell Cu_1_-Cu_2_ restricted to width of median band and located at juncture of this band with 2A. A black and short (approximately 1.5–2.0 mm) androconial patch at the distal third of 2A on the VHW.

Wing shape. FW triangular, costal margin convex, apex rounded, outer margin gently convex from apex to Cu_1_, tornus rounded, anal margin straight. VW costa slightly convex, apex rounded, outer margin crenulated, anal margin concave near tornus, remaining convex.

DFW. Greyish brown, darker along the costal and outer margins with a narrow ochre area on the first fourth of the costal margin length. Four dark brown to rufous bands, the former basal, dark brown (approximately 0.1 mm width) following the radial vein on its distal edge, the second submedian, dark rufous brown, from near the origin of R_1_ to 2A, crossing the discal cell near the middle, the third median, rufous and slightly concave, crossing the cell end from the origin of R_3_ across the base of Cu_1_ to 2A, curving distally near the inner margin, and the final band submarginal, dark brown, narrower and slightly crenulated from R_4_ to M_3_, rufous and posteriorly enlarged from M_3_ to 2A. Ocellus of the VFW observable through transparency.

DHW. Greyish brown. Four dark brown to rufous straight bands, the first basal, dark brown, short and tapered, the second submedian, dark rufous brown, from costal margin to anal margin, crossing the discal cell near the centre, the third median, rufous brown, from costal margin to anal margin, crossing the discal cell in its distal quarter, the final band submarginal, rufous and crenulated in each cell from Rs to anal margin, except Cu_1_-Cu_2_, following the contour of the outer margin, but widened and curved inwards in M_2_-M_3_ and to a lesser degree in M_3_-Cu_1_, wide and reddish orange from Cu_1_ to 2A, thinned from 2A to anal margin where it nearly reaches the median rufous band. Ocelli of the VHW observable through transparency.

VFW. Light greyish brown, bands similar to DFW. One developed black ocellus from M_1_ to anterior sixth in M_2_-M_3_, with a white pupil at the centre and a broad and yellow external ring, surrounded by a greyish brown area that extends posteriorly to the posterior half of M_3_-Cu_1_. Submarginal line dark brown, from R_4_ to anal margin, crenulated in R_4_-M_3_, remainder and straight, distally surrounded by a thin yellow line. Fringes dark brown.

VHW. Light greyish brown, bands similar to DHW. Three postmedian black ocelli, the anterior the smallest in Rs-M_1_, the second twice as wide as the first, from M_1_ to anterior third of M_2_-M_3_, and the posterior bigger, about three times wider than the second, ovoid, from the posterior third in M_3_-Cu_1_ to the edge of Cu_2_, all three with a white pupil at the centre and a broad and yellow external ring. A fourth minute black ocellus, with a yellow outer ring but with no pupil, located on the anal margin at the base of the postmedian brown band. Marginal dark brown, thin, and crenulated line from Sc+R_1_ to 3A. Fringes dark brown.

Male genitalia (Fig. 8). Tegumen dorsally flattened, trapezoidal, lateral posterior margin projecting ventrad as a short gnathos fused to the tegumen, subtriangular, nearly at right angle to uncus; uncus basally hairy, almost 10 times longer than wide, laterally apex curved downward; ventral arms of tegumen fused to anterior margin of tegumen; appendices angulares absent; anterior projection of saccus almost same length as uncus, dorsal arms of saccus combined with ventral arms from tegumen; valva sparsely hairy, basal two-thirds shaped vaguely as an elongated semi-circle, distal one-third rather narrow then widening to form a spatulate apex, distal half of valva in dorso-ventral view resembles propodus of a lobster (Decapoda: Nephropidae), but without the dactylus; aedeagus strongly curved upwards in lateral view, almost same length as valva, posterior portion opens latero-ventrally; cornuti absent.

**Figure 7.**
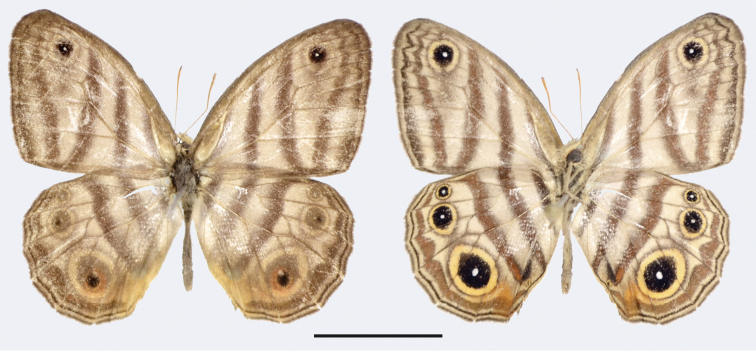
*Euptychia sophiae* sp. n. holotype male, dorsal (left) and ventral (right). FW length: 18.0 mm. DZUP collection. Photos by Thamara Zacca. Scale bar: 10 mm.

**Figure 8.**
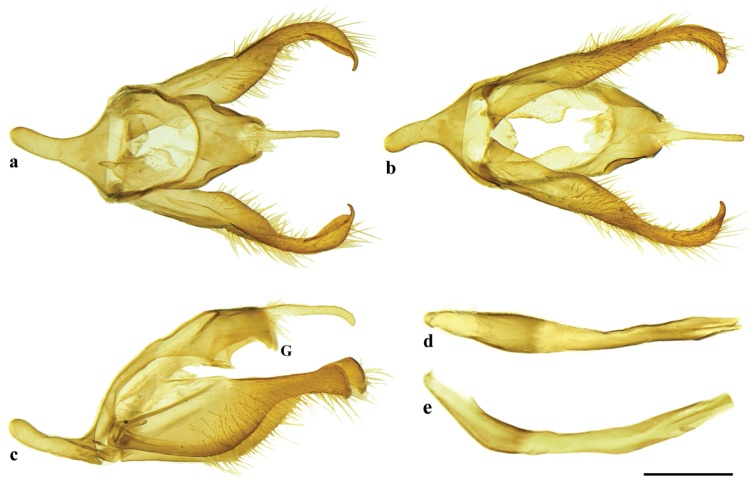
*Euptychia sophiae* sp. n. Genitalia of paratype male: **a** dorsal view **b** ventral view **c** lateral view (“G” indicates fused gnathos) **d** aedeagus, dorsal and **e** lateral views. DZUP collection. Photos by Diego R. Dolibaina. Scale bar: 0.5 mm.

FEMALE: Unknown.

#### Etymology.

The specific epithet honours T. Zacca’s niece, Laura Sophia. To prevent any future ambiguity, the name *sophiae* is considered to be a Latinised modern female noun in the genitive case.

#### Distribution and habitat.

**Figure 9.**
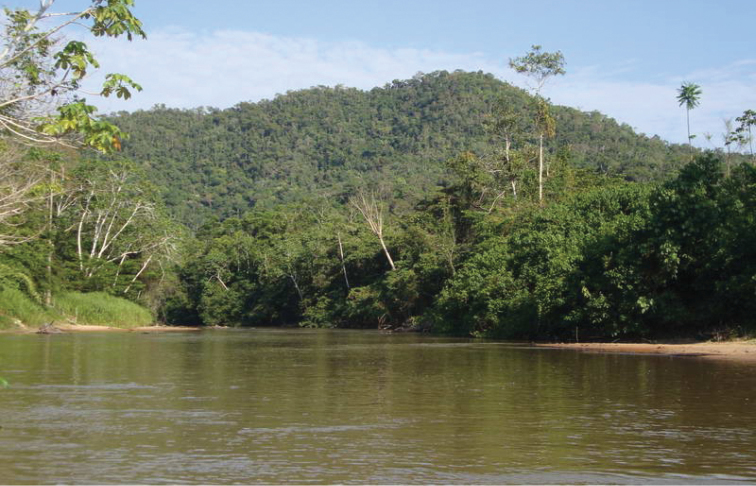
View from the Instituto Chico Mendes (ICMBio) research station at 200 m elevation of the river Moa and lowland tropical rainforest on the flatlands and mountain slopes of the northern Serra do Divisor National Park. Photo by Diego R. Dolibaina.

**Figure 10.**
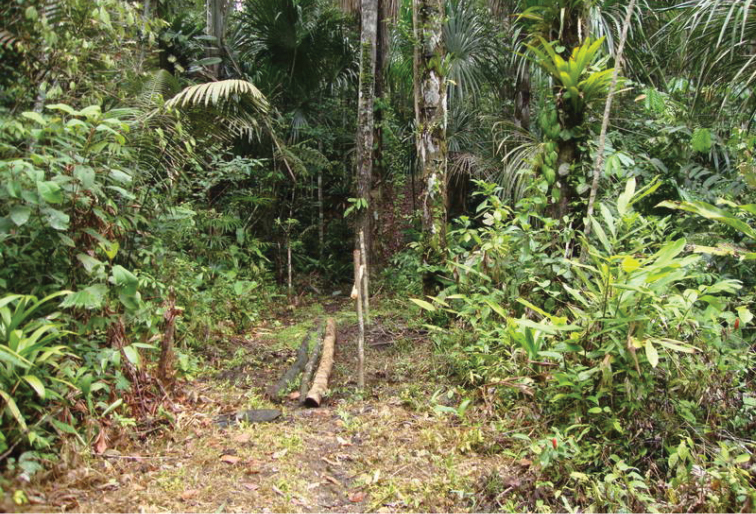
Seasonally inundated lowland tropical rainforest habitat with a predominance of *Mauritia* palms (Arecaceae) in the northern Serra do Divisor National Park. Photo by Diego R. Dolibaina.

This species is only known from the type locality in Serra do Divisor National Park (SDNP), Acre, in the extreme west of Brazil, and from across the border in the neighbouring department of Loreto, in north-eastern Peru. The Brazilian specimens of *Euptychia sophiae* were collected in forest characterized as submontane dense ombrophilous forest in a landscape of “terra firme” forest with patches of seasonally inundated areas with a predominance of palms in the genus *Mauritia* Linnaeus f. (Arecaceae) at about 200 m a.s.l. (see Figs 9, 10). A small north-south mountain range surrounds the low area in the western portion of the SDNP, with some hills as high as 600 m a.s.l. Only one hilltop was sampled (visible in Fig. 9) but no specimen of *Euptychia sophiae* was collected there, although other species of *Euptychia* were observed. Despite subsequent expeditions in June 2013 and August 2014 to the same area of the SDNP, with five and seven collectors respectively, we were unable to find additional specimens of *Euptychia sophiae*. Unfortunately, no behavioural notes were taken for this new species, but the three specimens known to us indicate a correlation with periods of average to below average rainfall, an observation similar to that made for *Euptychia attenboroughi*. Only future sampling will indicate whether these two species show a distinct preference for avoiding the months of highest annual precipitation.

#### Host plant.

Unknown.

## Discussion

Males of *Euptychia attenboroughi* and *Euptychia sophiae*, especially *Euptychia attenboroughi*, exhibit external wing pattern elements and wing morphology that are atypical for the genus, superficially recalling certain species in the genus *Chloreuptychia*. It was therefore important to support our generic classification for these two new taxa using more objective genetic analyses. Both new species are described in this genus on the basis of ML and Bayesian analyses of *Euptychia sophiae*, reinforced by the ML analysis performed in Nakahara et al. (2015): all analyses suggest that *Euptychia sophiae* should be a member of the monophyletic *Euptychia* clade with high support. Due to many external morphological similarities, including wing pattern and absence of a posterior projection of the tegumen in male genitalia, it is reasonable to consider *Euptychia sophiae* to be a sister species of *Euptychia attenboroughi*, which should therefore also be placed in *Euptychia*. The results indicate that the posterior projection of the tegumen is not shared by all members of the *Euptychia* clade, and that the absence of this character in these two species could be the result of secondary loss. Morphological evidence to support these two species in the *Euptychia* clade are: 1) presence of the forewing recurrent vein in the discal cell; 2) absence of basal swelling of the forewing cubital vein; 3) a relatively reduced humeral vein; 4) a developed 8^th^ tergite and sternite in the male abdomen; 5) presence of the sclerotized region of the 8^th^ abdominal segment in the female, located at the very basal side of the 8^th^ abdominal segment; 6) absence of the lateral sclerotisation of the 8^th^ abdominal segment of the female; 7) origin of the ductus seminalis at the posterior end of the ductus bursae; 8) absence of tibial spurs on the midleg and hindleg. States 1–7 are shared by all *Euptychia* species examined so far, and are rarely seen in other euptychiine butterflies. State 8 is shared by most species of *Euptychia* and those species that possess tibial spurs require further investigation regarding their classification. It is also noteworthy that within Euptychiina those *Euptychia* species whose early stage biology is known have both unique hostplants and larval characters (DeVries 1987, Beccaloni et al. 2008): known hostplants are non-seed plants in Selaginellaceae (Lycopodiophyta) and Neckeraceae (Bryophyta). States 1-8, hostplants, and early stage characters are possible apomorphic characters for *Euptychia*. Further in-depth study of euptychiine butterflies will hopefully determine this.

## Supplementary Material

XML Treatment for Euptychia attenboroughi

XML Treatment for Euptychia sophiae
